# Radiative cooling for passive thermal management towards sustainable carbon neutrality

**DOI:** 10.1093/nsr/nwac208

**Published:** 2022-09-30

**Authors:** Jun Liang, Jiawei Wu, Jun Guo, Huagen Li, Xianjun Zhou, Sheng Liang, Cheng-Wei Qiu, Guangming Tao

**Affiliations:** Wuhan National Laboratory for Optoelectronics and Optics Valley Laboratory, Huazhong University of Science and Technology, Wuhan 430074, China; Wuhan National Laboratory for Optoelectronics and Optics Valley Laboratory, Huazhong University of Science and Technology, Wuhan 430074, China; Department of Electrical and Computer Engineering, National University of Singapore, Singapore 117583, Singapore; MOE Key Laboratory of Thermo-Fluid Science and Engineering, School of Energy and Power Engineering, Xi’an Jiaotong University, Xi’an 710049, China; Department of Electrical and Computer Engineering, National University of Singapore, Singapore 117583, Singapore; Wuhan National Laboratory for Optoelectronics and Optics Valley Laboratory, Huazhong University of Science and Technology, Wuhan 430074, China; Key Laboratory of Education Ministry on Luminescence and Optical Information Technology, National Physical Experiment Teaching Demonstration Center, Department of Physics, School of Physical Science and Engineering, Beijing Jiaotong University, Beijing 100044, China; Department of Electrical and Computer Engineering, National University of Singapore, Singapore 117583, Singapore; Wuhan National Laboratory for Optoelectronics and Optics Valley Laboratory, Huazhong University of Science and Technology, Wuhan 430074, China; State Key Laboratory of Material Processing and Die & Mould Technology, School of Materials Science and Engineering, Huazhong University of Science and Technology, Wuhan 430074, China

**Keywords:** photonic structures, radiative cooling, energy saving and emission reduction, carbon neutrality

## Abstract

Photonic structures at the wavelength scale offer innovative energy solutions for a wide range of applications, from high-efficiency photovoltaics to passive cooling, thus reshaping the global energy landscape. Radiative cooling based on structural and material design presents new opportunities for sustainable carbon neutrality as a zero-energy, ecologically friendly cooling strategy. In this review, in addition to introducing the fundamentals of the basic theory of radiative cooling technology, typical radiative cooling materials alongside their cooling effects over recent years are summarized and the current research status of radiative cooling materials is outlined and discussed. Furthermore, technical challenges and potential advancements for radiative cooling are forecast with an outline of future application scenarios and development trends. In the future, radiative cooling is expected to make a significant contribution to global energy saving and emission reduction.

## Teaser

With global warming and excessive energy consumption, radiative cooling technology presents new opportunities for sustainable carbon neutrality as a zero-energy, eco-friendly cooling strategy.

## INTRODUCTION

Thermal comfort, heating ventilation and air conditioning (HVAC) systems requiring energy supply are widely used in space cooling, resulting in excessive consumption of fossil fuels and significant greenhouse gas emissions such as CO_2_ [1–7]. Massive CO_2_ and other greenhouse gas emissions have resulted in the rise of global temperature and climatic anomalies that significantly challenge the existence of life on Earth. In 2020, Antarctic temperatures exceed 20°C for the first time and extreme heat is expected to become the global temperature norm by 2050 [8]. Therefore, the development of energy-saving and ecologically friendly cooling techniques is critical to avoid these issues and achieve a sustained low-carbon lifestyle. However, emerging renewable-energy-driven cooling forms continue to require quite complex integrated systems to convert renewable energy into energy such as electricity, which may subsequently be utilized for cooling. Furthermore, these integrated systems, such as electric cooling driven by solar, water and wind energy, etc., have low conversion efficiency, occupy a large area and are limited by time and region [9–11]. To achieve a sustainable low-carbon paradigm, it is indispensable to develop novel cooling systems that are simple to integrate with a wide range of applications.

Compared with the aforementioned cooling methods, passive radiative cooling is of significant value owing to zero energy consumption, eco-friendliness, passiveness and sustainability, with reduced burning of fossil fuels and pollution emissions [12–18]. The heat of the object is transmitted to the low-temperature universe using electromagnetic waves through the atmosphere infrared (IR) window. The radiative heat exchanges with the space to reduce its temperature and achieve passive cooling. Researchers have identified that radiative cooling consistently lasts, with Earth’s surface continually emitting ∼100 PW of heat radiation into outer space [19]. Meanwhile, radiative cooling materials with judiciously designed photonic structures transmit or emit further middle-infrared (mid-IR) thermal radiation of the object, making a significant contribution to energy conservation and emission reduction.

In this paper, basic principles and key factors affecting the cooling effects of high-efficiency radiative cooling are discussed. The research progress in the passive radiative cooling (PRC) field over recent years is categorized into two main types. This includes mid-IR-transparent and mid-IR-emissive radiative cooling. Meanwhile, emerging radiative cooling technologies, alongside their challenges and future potential, are summarized. Finally, an ultimate blueprint for promoting carbon neutrality using radiative cooling technology has suitable prospects.

## RADIATIVE COOLING MECHANISM

**Figure 1.**
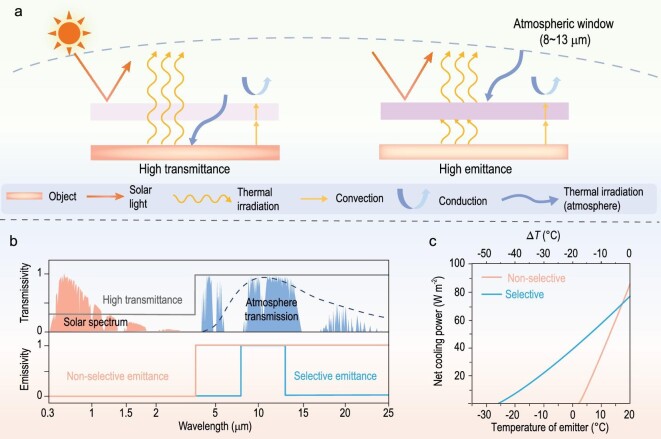
Mechanism of radiative cooling for passive thermal management. (a) Schematic heat-transfer process of the radiative cooling emitter. The light-purple and the dark-purple boxes are radiative cooling materials of high transmittance and high emittance, respectively. (b) Solar spectrum (orange-shaded area) and the atmosphere transmission spectrum in the mid-IR wavelength range (blue-shaded area). The transparency window of the atmosphere at 8–13 μm has a large spectral overlap with the blackbody radiative spectrum at typical ambient temperatures near 26.85°C (blue dashed curve). A spectral mid-IR-transparent surface (grey solid curve), selective (blue solid curve) and a non-selected mid-IR emitter (orange solid curve). (c) Functional relationship between net cooling power, temperature of emitter and Δ*T* (difference between ambient and emitter temperature) for the AM1.5 global tilt spectrum with irradiance of 964 W m^−2^, for ambient temperature 26.85°C, for the parasitic heat-transfer coefficient (conductive + convective) of 0 W m^−2^ K^−1^, for atmospheric transmittance in the zenith direction of 0.6 [36].

The working principle of PRC is shown in Fig. 1a. The fundamental principle is that a material surface spontaneously cools by reflecting sunlight [wavelengths (*λ*) of ∼0.3–2.5 μm] and radiating heat to the cold outer space through the atmosphere's long-wave infrared (LWIR) transmission window (*λ* of ∼8–13 μm) [15,20]. Furthermore, the structures of radiative cooling materials (purple boxes in Fig. 1a) vary, including multi-layer [21,22], porous [23] and composite structures [24–26]. Note that light scattering and thermal emission could be achieved simultaneously by some porous structures in mid-IR-emissive radiative cooling. For a clear sky, the transparency window of the atmosphere at 8–13 ${\rm{\mu m}}$ has a large spectral overlap with the blackbody radiative spectrum at typical ambient temperatures near 300 K [20,27]. As a result, the terrestrial radiation with wavelengths within the atmospheric window is highly transmissive to the entire atmosphere and the downward atmospheric heat radiation at these wavelengths could be negligible [28]. Conversely, the terrestrial thermal radiation outside the atmospheric window barely escapes the atmosphere. This is because electromagnetic waves outside this wavelength range cannot penetrate directly into space owing to the absorption of many gases, including N_2_, O_2_, CO_2_ and water vapour, etc. The atmosphere's downward thermal radiation is thus primarily at these LWIR wavelengths. To achieve efficient radiative cooling (e.g. deep sub-ambient temperature), a surface that absorbs neither solar nor downward atmospheric radiation should be designed [22,26,29–33].

Therefore, radiative cooling must achieve high reflection in the sunlight band, while radiative cooling in the LWIR band can be obtained by three approaches, i.e. high transmittance, non-selective and selective emittance, respectively, as shown in Fig. 1b. For high mid-IR-transparent radiative cooling, the materials typically possess a thickness of <150 μm to maintain sufficient transmittance, resulting in restrictions in solar reflection and mechanical properties [34,35].

For mid-IR emittance, two types of effective radiative cooling exist. One is a non-selective emitter, with a nearly uniform emissivity of 1 in the infrared wavelength spectrum from 2.5 to 25 μm, like the blackbody. Conversely, the selective emitter has an emissivity close to 1 in the thermal infrared range from 8 to 13 μm, and almost zero outside the band. Meanwhile, net cooling power is an important evaluation of the radiative cooling material’s performance. Based on the non-selective emitter characteristics, the emitter will also strongly absorb heat radiation from the atmosphere downward, in addition to the wavelength of the whole transmitting heat-source infrared band, owing to the reciprocity between emission and absorption. Thus, when the object’s operating temperature is higher than the ambient temperature, the non-selective emitter has a higher net cooling power. This is because the radiation is stronger than the radiation absorbed from the atmosphere outside the transparent window. Conversely, if the object is operating at a temperature below or close to the ambient temperature, the power absorbed by atmospheric radiation outside the transparent window can offset the thermal radiation. Therefore, the selective emitter has a higher net cooling power. For example, when Δ*T* = 17°C (the difference between ambient and emitter temperature), the difference in net cooling power is 42 W m^−2^. In this scenario, the selective emitter is referred to as a radiative cooling material because it ignores downward atmospheric thermal radiation outside the atmospheric transparent window, lowering the temperature of the heat source. The steady selective cooler temperature is significantly lower than the broadband emitter under the conditions of zero solar radiation and non-radiative heat exchange (see Fig. 1c) [36]. However, Jonghwa Shin's group verified that a selective emitter can be subtly designed to outperform a non-selective emitter at any temperature below the air temperature [37].

In addition to the object's thermal radiation power, atmospheric radiation and non-radiative heat transfer of the surrounding medium may impact PRC performance. To accurately evaluate PRC performance, the thermal insulation substrate and convection shield are generally used to avoid conduction and convection heat transfer, respectively [38]. Compared to high mid-IR-transparent radiative cooling materials, mid-IR-emittance materials not only relax the limitation on thickness but also alleviate the reverse effect of atmospheric radiation on objects at higher ambient temperatures.

## MID-IR-TRANSPARENT RADIATIVE COOLING

Radiation plays a dominant role in the heat loss of thermal emitters, like the human body, which emits thermal radiation in the mid-IR range [35,39–42]. Considering the IR optical property, the novel radiative cooling technique features energy-efficient thermal management. In a typical indoor scenario below human body temperature, radiative cooling in the infrared wavelength range accounts for >50% of total human body heat loss [43]. However, the body’s radiation heat dissipation is hindered by general clothing for clothing is necessary. Hence, for human skin with high mid-IR emissivity, the cooling effect is enhanced by promoting thermal radiation from the body to the environment through materials with high mid-IR transmission. Furthermore, we wear textiles indoors and must consider visible opacity for textiles. In other words, the mid-IR transparency coupled with being opaque to visible light is a practical solution to achieving enhanced heat dissipation for the human body [44,45]. Table 1 lists reported studies of PRC performance with high mid-IR transmittance.

**Table 1.** List of mid-IR-transparent radiative cooling studies of previously reported performance.

Reference	Structure and material	Thickness (μm)	Thermal transmittance	Cooling power (W m^−2^)	Temperature decrease (°C)	Solar intensity (W m^−2^)	Ambient temperature (K)	Energy saving	Remark
[46]	PE nanofibers	300	0.972	23	/	/	300	40%	Sim.
[47]	NanoPE	12	>0.9	/	2	/	296	7%–45%	Exp.
[48]	Nylon–nanoPE	/	0.921	/	2.5–6.3	/	298	/	Exp.
[49]	ZnO + nanoPE	150	∼0.8	200	10	900	300	/	Exp.
[35]	NanoPE	450	>0.7	/	2.3	/	296.5	20.1%	Exp.
[43]	Inorganic pigment NPs–mixed PE	100	∼0.8	/	1.6–1.8	/	298	>10%	Exp.
[50]	NanoPAN	720	>0.7	/	3	960	298	/	Exp.
[51]	NanoPE–Al	∼40	∼0.7	/	4	/	300	/	Exp.

Sim. = simulations, Exp. = experiments.

**Figure 2.**
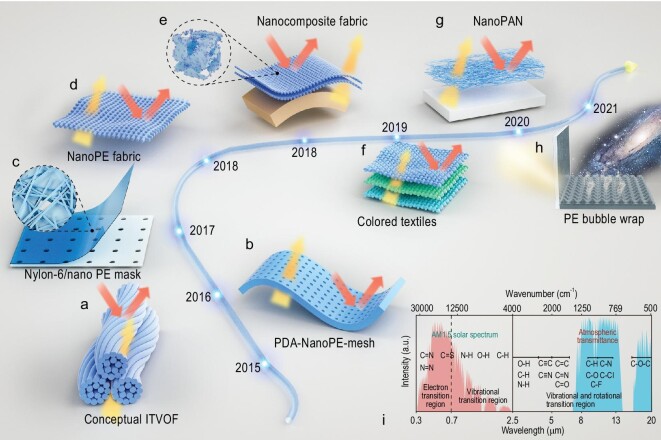
Roadmap of mid-IR-transparent materials for passive radiative cooling. (a) Theoretical framework to design IR-transparent fabric. (b) Schematic of polydopamine and nano-porous polyethylene (PDA-NanoPE-mesh) with performance comparable to that of cotton. (c) Diagram of face masks with electrospun nylon-6 nanofibers on needle-punched nanoPE substrate. (d) Schematic of nanoPE fabric with high mid-IR transparency, visible opacity and good wearability. (e) ZnO NPs embedded nano-porous PE fabric. (f) Schematic for the colouration of radiative cooling textiles, which is made by mixing IR-transparent inorganic pigment nanoparticles with PE. (g) Direct radiative cooling using IR-selective transparent covers. (h) Schematic of highly IR-transparent PE bubble warp to block ambient convective heat transfer and ensure the radiative heat transport from objects to outer space. (i) The selection of functional groups for high emittance and low solar absorption.

Initially, an IR-transparent visible-opaque fabric (ITVOF) was theoretically designed to advance radiative cooling from the perspective of IR transmittance [46]. The ITVOF was developed using synthetic polymer fibers, which were featured by intrinsically low IR absorptance and structured to minimize IR reflection via weak Rayleigh scattering while maintaining visible opaqueness via strong Mie scattering (see Fig. 2a). Furthermore, Hsu *et al.* first processed a textile using nano-porous polyethylene (nanoPE) with interconnected pores of 50–1000 nm in diameter [47]. The material is transparent to mid-IR human body radiation because of the gap between the pore sizes and IR wavelength. The pore sizes are comparable with the wavelength of visible light (400–700 nm), contributing to the opaqueness to human eyes. Mid-IR-transparent material and nanoscale pores are integrated for selective spectral control (see Fig. 2b). In addition, nanoPE was also introduced to nanofiber face masks as a substrate to enhance user thermal comfort [48]. The nanofiber/nanoPE model system can efficiently prevent particulate matter pollution with low resistance and is simultaneously transparent to human body thermal radiation. Regardless that nanoPE with embedded pores has revealed powerful thermal management via the extreme IR optical property, its non-woven nature may obstruct further applications as wearable scalable textiles (see Fig. 2c).

To improve the wearable properties of nanoPE, Peng *et al.* developed a large-scale fabrication technique involving uniform continuous nanoPE with cotton-like softness [49]. NanoPE microfibers can be mass-produced via a fiber-extrusion process. Concerns of necessary wearable properties, such as air permeability and touch comfort, can be addressed by the knitted/woven fabric. Visible opacity and mid-IR transparency were maintained to lower human skin indoor temperature by 2.3°C (corresponding to >20% indoor energy savings). The nanoscale pores allowed the microfiber bending motion and ensured PE materials are soft to wear (see Fig. 2d). In the outdoor scenario, a ZnO nanoparticle-embedded nanoPE (ZnO-PE) was demonstrated as an effective radiative cooling textile [35]. The ZnO-PE endowed >90% solar irradiance reflection and high transmission of thermal radiation by spectral selectivity. Compared with normal textiles, this can experimentally prevent simulated skin from overheating by 5–13°C (corresponding to a cooling power of >200 W m^−2^). The lack of textile colour tunability maintains a grand impediment to large-scale commercialization (see Fig. 2e).

To advance further, Cai *et al.* utilized inorganic nanoparticles as a colouring component for coloured and mid-IR-transparent textiles [43]. The dilemma between visible and IR optical properties was resolved using pigment nanoparticles, which were almost transparent in the IR region while reflecting certain visible colours via optimized concentration and size. Colour stability was maintained after >100 washing cycles with a radiative cooling ability of 1.6–1.8°C (see Fig. 2f). Solar scattering is another critical factor affecting the radiative cooling performance. It was reported that nanofibers with ellipsoidal beads exhibited the most efficient solar scattering because of additive dielectric resonances between the ellipsoidal and cylindrical geometries [50]. The nanofibers decreased the amount of material used to reach >95% solar reflectance while maintaining >70% IR transmittance (see Fig. 2g). To fill the gap between efficient radiative cooling and cost-effectiveness, PE bubble wrap and aluminium foils were combined to process a facile and low-cost radiative cooling film [51]. An average sub-ambient temperature reduction of 4°C was achieved (see Fig. 2h). Figure 2i)shows the absorption of polymer chemical bonds in the wavelength range from 0.3 to 20 μm [52,53]. In addition to thickness constraints, the selection of materials for mid-IR-transparent radiative cooling requires a reduction in the content of functional groups that absorb sunlight and mid-IR radiation to obtain improved performance. It is worth noting that recent work in mid-IR-transparent radiative cooling has achieved energy savings of almost 40% [41], 7%–45% [47], 20.1% [49] and ≥10% [43] under different environmental conditions.

## MID-IR-EMISSIVE RADIATIVE COOLING

Many centuries ago, tropical and subtropical areas could properly use night radiative cooling to cool buildings and freeze and desalinate water. In early academic research, there were some natural or artificial materials, such as white pigment [54,55], high-polymer film [56], silicon oxide film and others [57,58]. Although these radiative cooling materials can represent certain radiative characteristics, the radiative rate is low. They lack the micro/nano optical design of materials, which explains why early cooling devices failed to achieve a stable cooling effect significantly lower than the ambient temperature [56,59,60].

**Figure 3.**
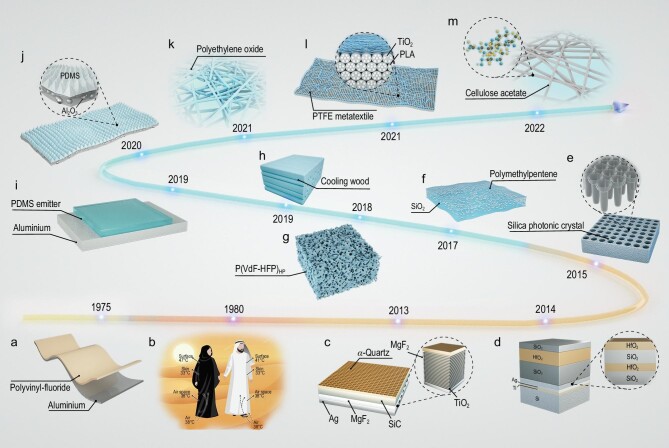
Roadmap of mid-IR emissive materials for passive radiative cooling. (a) Diagram of coating a metal surface with cheap plastic materials. (b) Enhanced convection of air beneath the black robes carries this heat away before it reaches the skin making it just as effective as the white robes. (c) Diagram of ultra-broadband metal–dielectric photonic structures. (d) Diagram of HfO_2_–SiO_2_ photonic film. (e) Diagram of visibly transparent silica photonic crystal. (f) Diagram of the scalable-manufactured glass–polymer hybrid metamaterial. (g) Diagram of hierarchically porous polymer coatings. (h) Diagram of the cooling wood structure. (i) Schematic diagram of the planar PDMS/metal thermal emitter. (j) Artificially fabricated radiative coolers based on the fluff structure of *Neoregelia gigas*. (k) Effective scattering diagram of micro- and nanostructures of the es-PEO film. (l) Hierarchical-morphology metafabric for scalable radiative cooling. (m) Diagram of the intrinsic molecular vibrations and porous structure of the cellulose acetate (CA) film.

Previous work in daytime radiative cooling has sought to achieve radiative cooling by coating a metal surface with cheap plastic materials (see Fig. 3a) [61]. During daytime, such a design allows a selective surface to be more effectively cooled when exposed to a clear sky. Furthermore, Shkolnik *et al.* investigated Bedouin people who wear black robes in the hot desert, while studying daytime PRC (see Fig. 3b) [62]. Results showed that wearing a black robe in this environment cools body temperature. It is difficult for natural materials to achieve PRC by reflecting sunlight and emitting heat radiation. In contrast, micro-nano optical materials represented by photonic crystals and metamaterials could achieve high reflection solar radiation and high emission infrared radiation from thermal sources using precise optical design and adjustment. Table 2 lists reported studies of PRC performance with high mid-IR emission.

**Table 2.** List of mid-IR-emissive radiative cooling studies of previously reported performance.

Reference	Structure and material	Thickness (μm)	Solar reflectance	Thermal emittance	Cooling power (W m^−2^)	Temperature decrease (°C)	Solar intensity (W m^−2^)	Ambient temperature (K)	Energy saving	Remark
[21]	Quartz–SiC–MgF_2_–TiO_2_–Ag	1.8	0.965	/	105	/	964	300	/	Sim.
[15]	HfO_2_–SiO_2_–Ag	1.6	0.97	/	40.1	4.9	850	/	/	Exp.
[63]	Si_3_N_4_–Si–Al	525.3	/	0.962	/	13	1000	290	20%	Exp.
[64]	TPX + SIO_2_ spheres–Ag	50	0.96	0.93	93	/	900	/	/	Exp.
[65]	Porous PVDF	300	0.96 ± 0.03	0.97 ± 0.02	96	6	890	/	/	Exp.
[66]	PDMS–Al	150	/	/	120	∼2–9	∼853.5	/	/	Exp.
[67]	PDMS + Al_2_O_3_ spheres	500	∼0.95	>0.96	∼90.8	5.1	862	305	/	Exp.
[68]	PEO–nanofiber-based film	500	96.3	0.78	110	7	900	290	30%	Exp.
[69]	PTFE–PLA + TiO_2_ spheres	500	0.924	0.945	/	4.8	∼650	295	**/**	Exp.
[70]	Porous CA	/	0.974	0.92	110	12	∼825	290	/	Exp.

Sim. = simulations, Exp. = experiments.

Traditional photonic crystals are designed with a periodic structure to accurately control light. Fan *et al.* first proposed a metal–dielectric photonic structure capable of radiative cooling under daytime outdoor conditions [21]. The structure behaves as a broadband mirror for solar light, while simultaneously emitting strongly in the mid-IR within the atmospheric transparency window and achieving a net cooling power of >100 W m^−2^ at ambient temperature (see Fig. 3c). Further, Raman *et al.* designed a cooling device using the seven layers of a HfO_2_ and SiO_2_ integrated photonic solar reflector and thermal emitter, exhibiting 97% strong solar reflection and selectively strong emission in the transparent atmospheric window [15]. This suggests that the photonic method can be tailored to achieve energy efficiency and realize energy conservation and emissions reduction (see Fig. 3d). With the development of new energy technologies, solar photovoltaic panels became representative, although this was accompanied by one problem—absorbing a significant amount of solar radiation for power generation, resulting in overheating at the bottom. Based on silica photonic crystals, Fan *et al.* designed a visibly transparent thermal blackbody [63]. When placed on a silicon absorber under sunlight, such a blackbody preserves or even slightly enhances sunlight absorption, although it reduces the temperature of the underlying silicon absorber by as much as 13°C owing to radiative cooling (see Fig. 3e). This research has significant implications for improving efficiency and extending the lifetime of solar photovoltaic panels.

Periodic photonic structures are extremely challenging with regard to process production and industrial cost. Thus, the design of metamaterials with randomly distributed micro and nanoparticles on the inside and surface has major scientific and social significance for the radiative cooling field through accurate optical structure design, Mie theory and the difference in the refractive index and chemical bond of materials. Thus, it obtains high sunlight reflection and high emission of infrared waves from a heat source.

Currently, metamaterial morphology is represented by thin films and fibers. Many researchers have developed different thin-film radiative cooling devices. Among them, Yin *et al.* randomly embedded resonant polar dielectric microspheres in a polymeric matrix, forming a metamaterial fully transparent to the solar spectrum while having an infrared emissivity of >0.93 across the atmospheric window [64]. When backed with a silver coating, the metamaterial shows a noon-time radiative cooling power of 93 W m^−2^ under direct sunlight (see Fig. 3f). Yang *et al.* presented a simple, inexpensive and scalable phase-inversion-based method for fabricating hierarchically porous poly (vinylidene fluoride-co-hexafluoropropene) [P(VdF-HFP)_HP_] coatings with excellent passive daytime radiative cooling capability [65]. This substrate-independent material has solar reflectivity of ≤0.96 ± 0.03, LWIR emissivity of ≤0.97 ± 0.02, sub-ambient temperature drops of ∼6°C and cooling powers of ∼96 W m^−2^ (see Fig. 3g). The cooling effect, featured by technological simplicity and industrial affordable cost, is of major scientific and social significance regarding global warming and building cooling. Furthermore, Hu *et al.* designed a cooling wood structure by a process of complete delignification and densification of wood [66]. The delignified and mechanically pressed wood also delivers mechanical strength and toughness, respectively. It could reflect solar radiation and emit strongly in LWIR, resulting in continuous sub-ambient cooling during a full day. It is worth noting that cooling wood can achieve energy savings of between 20% and 60% (see Fig. 3h). In addition, Gan *et al.* designed an inexpensive planar polydimethylsiloxane (PDMS)/metal thermal emitter thin-film structure [67], fabricated using a fast solution coating process, scalable and suitable for large-area manufacturing (see Fig. 3i). This also achieves stable cooling and a temperature reduction of 11°C. Based on the phenomenon that natural organisms typically survive in a hot environment, Fan *et al.* designed a photonic film consisting of a micropyramid-arrayed polymer matrix with random ceramic particles fabricated with high throughput achieving high reflection, high emission and large-scale production [68]. Additionally, the film exhibits hydrophobicity, superior flexibility and strong mechanical strength, which is promising for thermal management in various electronic devices and wearable products (see Fig. 3j).

Similarly, much research has reported radiative refrigerating metamaterials in the form of fibers, primarily for personal thermal management (PTM). As global warming intensifies, the harm of solar radiation to human skin significantly increases. Most people opt to use active cooling devices such as air conditioners and fans. Therefore, it is necessary to establish a balance between human body thermal comfort, energy saving and emission reduction. Zhu *et al.* proposed a hierarchically designed polymer nanofiber-based film [69], produced by a scalable electrostatic spinning process and enabled selective mid-IR emission and effective sunlight reflection, showcasing excellent all-day radiative cooling (see Fig. 3k). This layered structure has a 78% selective emissivity in the wavelength range from 8–13 μm owing to the C–O–C (1260–1110 cm^−1^) and C–OH (1239–1030 cm^−1^) bonding and the design of nanofibers with a controlled diameter enables a high reflectivity of 96.3% in the wavelength range of 0.3–2.5 μm. Tao *et al.* designed a hierarchical morphology of the randomly dispersed scatterers throughout the metafabric (see Fig. 3l) and large-scale woven metafabrics provide emissivity of 94.5% in the atmospheric window and reflectivity of 92.4% in the solar spectrum [70]. The human body cooling test shows an excellent cooling effect; when covered by metafabric, it could be cooled down ∼4.8°C lower than that covered by commercial cotton fabric and this is waterproof, showing good air permeability. The fabric and its garments can be effectively applied to PTM and have significant potential in logistics, transportation and light protection products. To further advance, Zhu *et al.* hierarchically designed a radiative cooling film based on abundant and eco-friendly cellulose acetate molecules provides effective and passive protection in a versatile way to various forms/scales of ice under sunlight (see Fig. 3m). The intrinsic vibrations of the molecular bonding guarantee the cellulose acetate (CA) film will exhibit broadband and high mid-IR emissivity, which is desirable for high-performing large-scale cooling [71]. This work demonstrates radiative cooling as a promising avenue toward effective sustainability. Previously research on PRC has achieved excellent cooling performance. Particularly, researchers report that their cooling devices achieve energy savings of almost 20% [63] and 30% [30] without producing any form of emissions.

## SUMMARY AND OUTLOOK

It has been established that peak CO_2_ emissions and subsequent carbon neutrality are predominant global challenges for future societal development. The former refers to a point in time when CO_2_ emissions cease to peak and then gradually fall back. Furthermore, it is necessary to achieve relatively close to ‘zero’ CO_2_ emissions, e.g. through energy saving and emissions reduction, which constitutes carbon neutrality. It is anticipated that radiative cooling techniques will play an essential role in PTM owing to net-zero energy consumption and environmentally friendly characteristics. In this study, recent progress on advanced radiative cooling technologies was reviewed and classified according to radiative cooling mechanisms. Although radiative cooling has opened new avenues for energy saving and pollution reduction, challenges still go together with opportunities from device to system.

First, selective radiative materials must be further explored. Modifying surface morphological structures to control IR transmittance or emittance is pivotal for efficient spectral selectivity. However, optimizing the structure profile without compromising scalable fabrication remains a challenge. It is necessary to develop low-cost interfacial microstructures to achieve high-performance radiative cooling. From a molecular perspective, it is applicable to select functional groups to control optical properties in different spectral regions. The additive dyestuff may affect the spectral characteristics of radiative materials and it is difficult to balance colour and cooling performance. One promising direction is to control the optical properties in IR and visible light regions simultaneously using microstructures to achieve structural colour. The wavelength conversion technique may also present another promising paradigm.

Moreover, systematic performance of radiative cooling techniques is critical for broader applications in thermal management. Potential exists for the combination of radiative coolers with other new energy facilities to improve energy-conversion efficiency and maintain stable work conditions. Low efficiency is considered a long-standing challenge for solar cells. The use of radiative coolers can reflect solar radiation (1.1–2.5 μm) to provide an excellent thermal environment. Radiative coolers could also enhance radiative and conductive heat dissipation to address the challenge of solar cell low thermal conductivity. With the development of the thermoelectric generator, power density increases, leading to severe thermal management problems. The integration of a radiative cooler can improve cooling performance. Nevertheless, when considering the full-day power generation of thermoelectric devices, higher power density may require other cooling pathways combined with radiative coolers. Meanwhile, deployment of thermoelectric generators mitigates energy problems in extreme environments such as deserts.

In addition to energy-conversion systems, harvesting water from the atmosphere is another dramatic application for radiative cooling. It is necessary to speed up droplet nucleation on the radiative cooler surface and remove the collected water as soon as possible. Therefore, it is possible to equip the radiative cooler with hydrophilic and directional slippery surfaces. When encountering highly arid conditions, cooling performance should be improved. This significantly contributes to the collection of fresh water in desert environments. Radiative cooling has also been deployed in space engineering, which imposes particularly strict conditions regarding energy consumption, and radiative cooling paints and woods provide a lightweight approach to the reduction of energy consumption.

**Figure 4.**
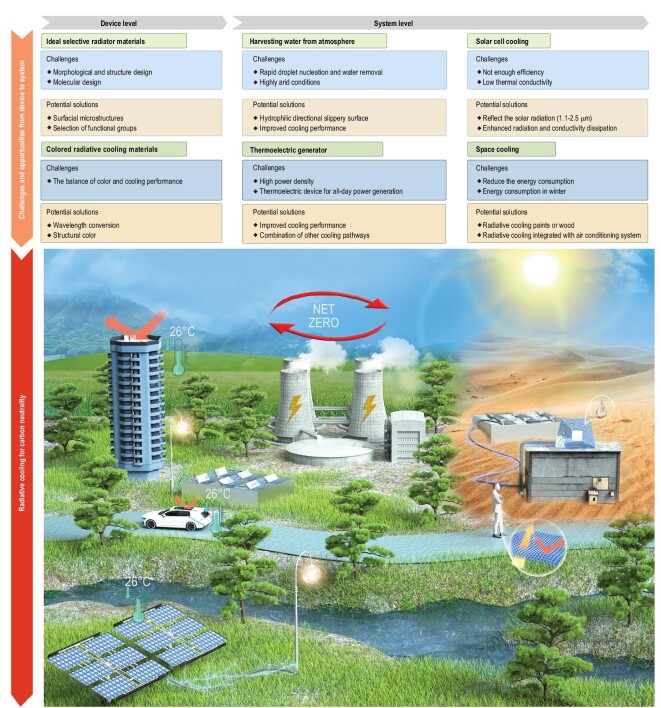
Challenges and potential solutions for radiative cooling from materials to system perspectives and radiative cooling toward sustainable carbon neutrality.

Radiative cooling sheds new light upon carbon peak and neutrality (see Fig. 4). Radiative coolers also present a passive strategy for various vehicles, lowering gas exhaust emissions. Furthermore, part of the energy consumption required to cool buildings and the human body will likely be substituted by the radiative cooler to satisfy energy-conservation goals. Notably, radiative cooling spectrum selectivity will accelerate solar energy technology progress. Additionally, the utilization of super-white paints for radiative cooling is gaining popularity. However, radiative cooling technology performance is limited by the environment, especially under high-humidity conditions (tropical or subtropical regions). This is because the atmospheric window shrinks with increasing humidity [72]. Other passive cooling technologies such as seasonal ice storage are also obstructed by the effects of humid environments. It is worth noting that the salinity difference system proposed by Chiavazzo *et al.* is completely unaffected by atmospheric humidity values [73]. However, this system is limited by intense solar radiation. Furthermore, a tandem passive cooling of evaporation and radiation exhibits excellent cooling performance under various conditions of weather/climate [74,75]. Therefore, by combining radiative cooling techniques with passive cooling techniques such as salinity difference systems and evaporative cooling, the implementation of energy-saving strategies in space cooling and human thermal management systems is expected to extend future application scenarios.
